# Supplementary material for: GOAT: efficient and robust identification of gene set enrichment
Source: Commun Biol. 2024 Jun 19;7:744. doi: 10.1038/s42003-024-06454-5 (PMC11187187; doi:10.1038/s42003-024-06454-5)
Supplement: Supplementary file 2 — Supplementary Information [file 42003_2024_6454_MOESM2_ESM.pdf]

Supporting Information

GOAT: efficient and robust identification of gene set enrichment

Frank Koopmans

Department of Molecular and Cellular Neurobiology, Center for Neurogenomics and Cognitive Research, Amsterdam Neuroscience, VU University, 1081HV Amsterdam, The Netherlands.

Table of Contents

Supplementary Figure 1. GOAT gene scores and gene set null distributions from the Colameo et al. mass spectrometry dataset. Related to Figure 2.

Supplementary Figure 2. Computation time for each algorithm on simulated datasets of various sizes.

Supplementary Figure 3. Simulations for GSEA using gene lists and gene sets of various sizes. Related to Figure 2.

Supplementary Figure 4. RMSE of GOAT and GSEA under null hypothesis. Related to Figure 2.

Supplementary Figure 5. Characterization of real-world datasets that were used for ROC analyses. Related to Figure 3.

Supplementary Figure 6. Gene set p-value distributions from various simulated gene set collections, based on the Colameo RNA-sequencing dataset. Related to Figure 3.

Supplementary Figure 7. All ROC simulations using the Colameo RNA-sequencing dataset as input gene list. Related to Figure 3.

Supplementary Figure 8. Analogous to Supplementary Figure 7, but here using the Higginbotham mass spectrometry dataset as input. Related to Figure 3.

Supplementary Figure 9. Analogous to Supplementary Figure 7, but here using the Sahadevan RNA-sequencing dataset as input. Related to Figure 3.

Supplementary Figure 10. Analogous to Supplementary Figure 7, but here using the Wingo mass spectrometry dataset as input. Related to Figure 3.

Supplementary Figure 11. Proportion of gene sets where iDEA did not return a valid p-value. Related to Figure 4.

Supplementary Figure 12. Characterization of missing data in iDEA. Related to Figure 4.

Supplementary Figure 13. Comparison of GSEA gene set p-values to iDEA and GOAT. Related to Figure 4.

Supplementary Figure 14. Application of alternative GSEA and iDEA configurations to real-world data. Related to Figure 4.

Supplementary Figure 15. Comparison of GOAT top-hits with GSEA and ORA in application to real-world data. Related to Figure 4.

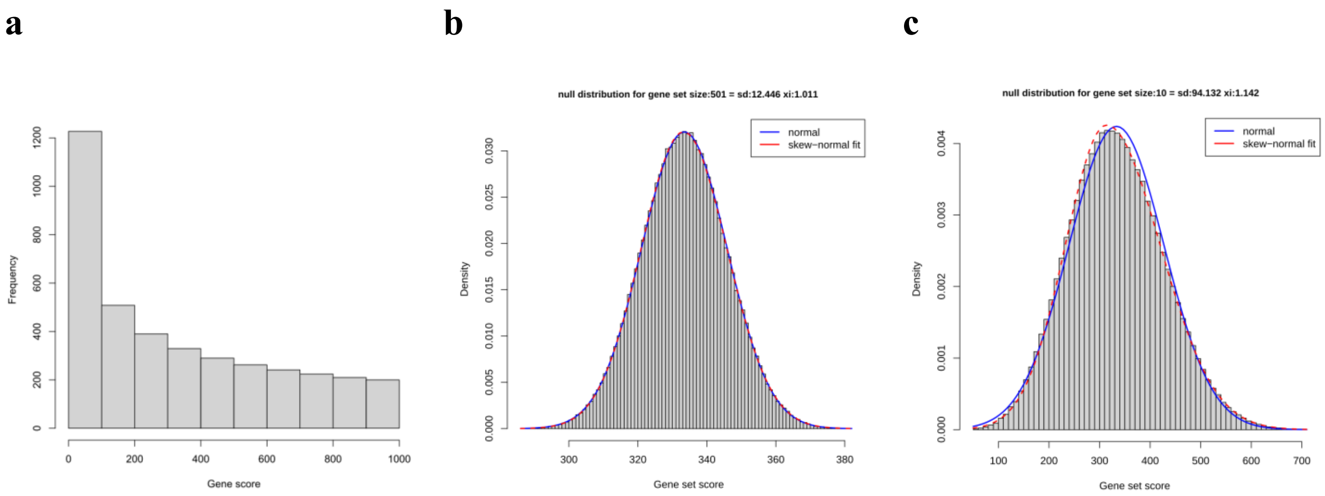

Supplementary Figure 1. GOAT gene scores and gene set null distributions from the Colameo et al. mass spectrometry dataset. Related to Figure 2.

a) A histogram of the gene scores, computed from $rank^2$ transformed gene p-values. **b**) Null distribution of gene set scores generated for gene sets that many genes (501). **c**) Analogous to B, but for a small gene set of only 10 genes. The blue line illustrates the fitted normal distribution. The dashed red line illustrates the fitted skew-normal distribution which describes the distribution generated from small genesets (c) better than a normal distribution (blue line).

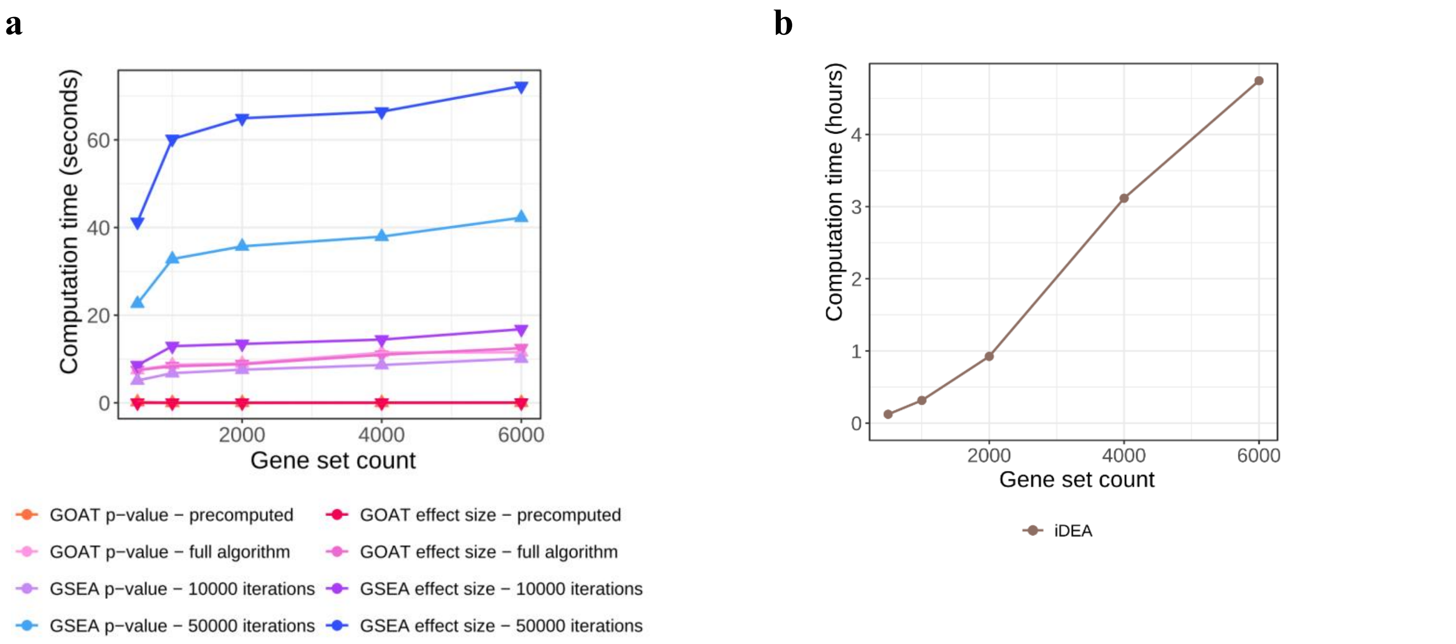

Supplementary Figure 2. Computation time for each algorithm on simulated datasets of various sizes.

We generated a random gene list and evaluated the computation time needed to test gene set significance of 500, 1000, 2000, 4000 or 6000 randomly selected Gene Ontology (GO) terms. A high performance workstation with AMD 3900X 12-core, 24-thread processor was used. Benchmarking analyses were performed on a GNU/Linux operating system.

a) GOAT completed each analysis within 1 second when using the precomputed null distributions. Changing the fGSEA R package parameter 'nPermSimple' to 10 000 or 50 000 (default is 1000) improved accuracy (see further Supplementary Figure 3), but also impacted computation time. Regardless, computation times in the order of seconds up to a few minutes do not limit practical use. **b**) Analogous to (a), we applied the iDEA algorithm and observed computation times in the order of hours even when using a fast workstation computer.

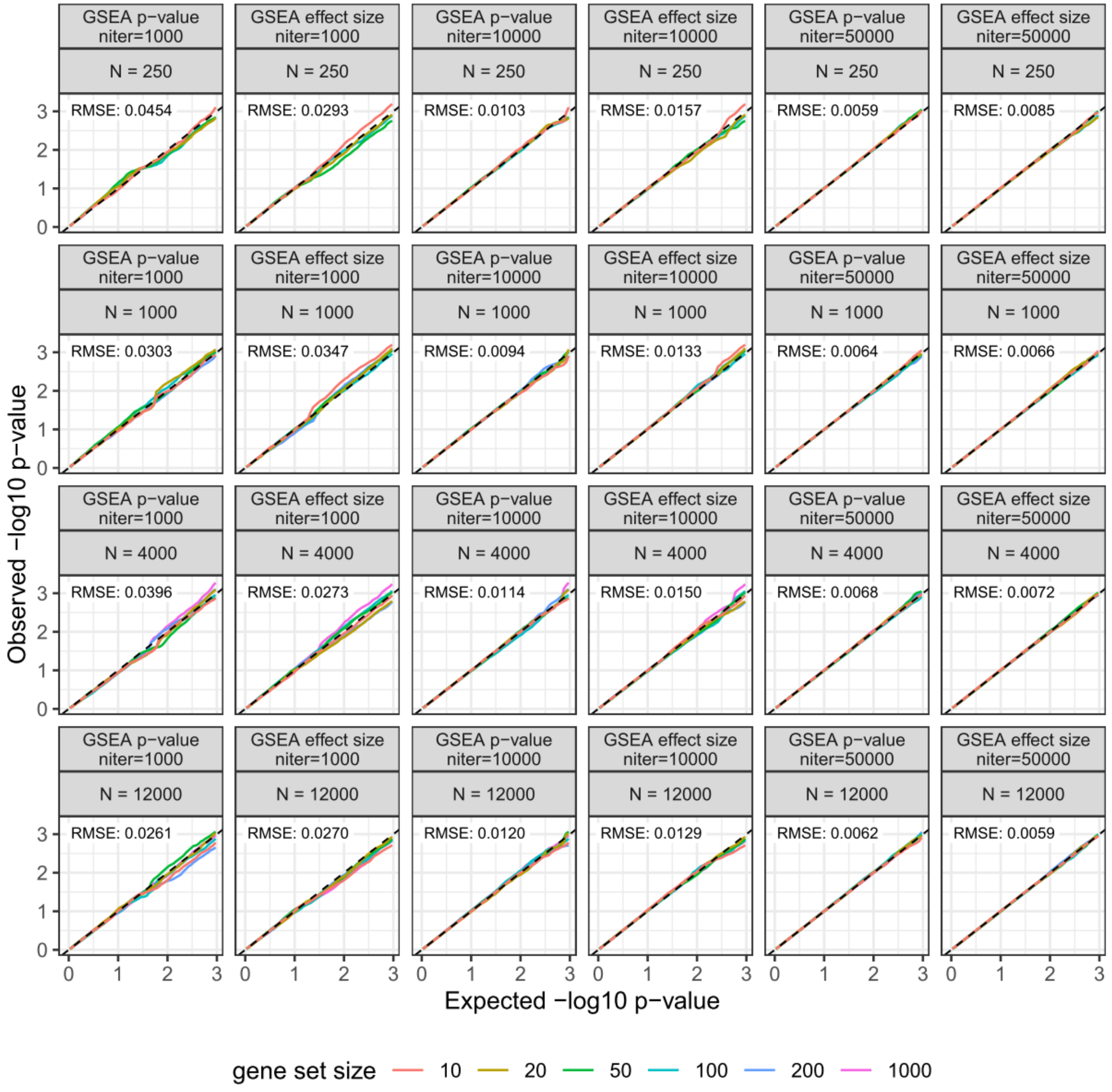

Supplementary Figure 3. Simulations for GSEA using gene lists and gene sets of various sizes. Related to Figure 2.

Analyses analogous to Figure 2, but here describing 3 different settings for GSEA to control the number of permutations (fGSEA R package, parameter 'nPermSimple' , here labeled as 'niter'). While increasing from default 1000 iterations to 10 000 is an improvement, further accuracy gains are observed at 50 000 iterations.

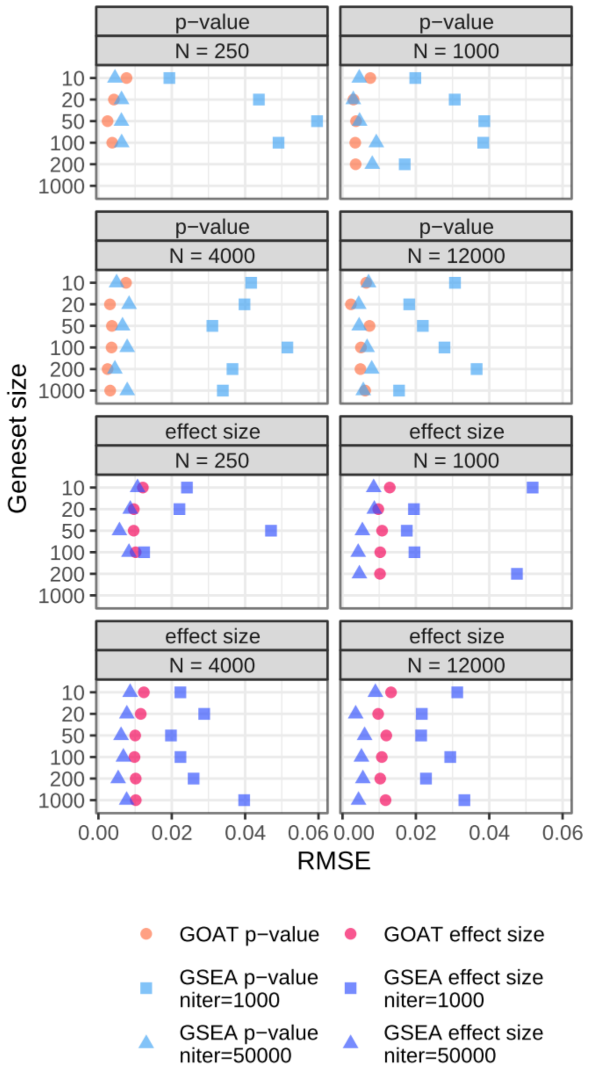

Supplementary Figure 4. RMSE of GOAT and GSEA under null hypothesis. Related to Figure 2.

Overview of all Root Mean Square Error (RMSE) values (x-axis) observed in null hypothesis simulations. The y-axis shows respective gene set sizes that are depicted as color-coded lines in Figure 2. Panels indicate gene list length, matching the rows in Figure 2. GSEA (fGSEA R package) benefits from increased number of iterations.

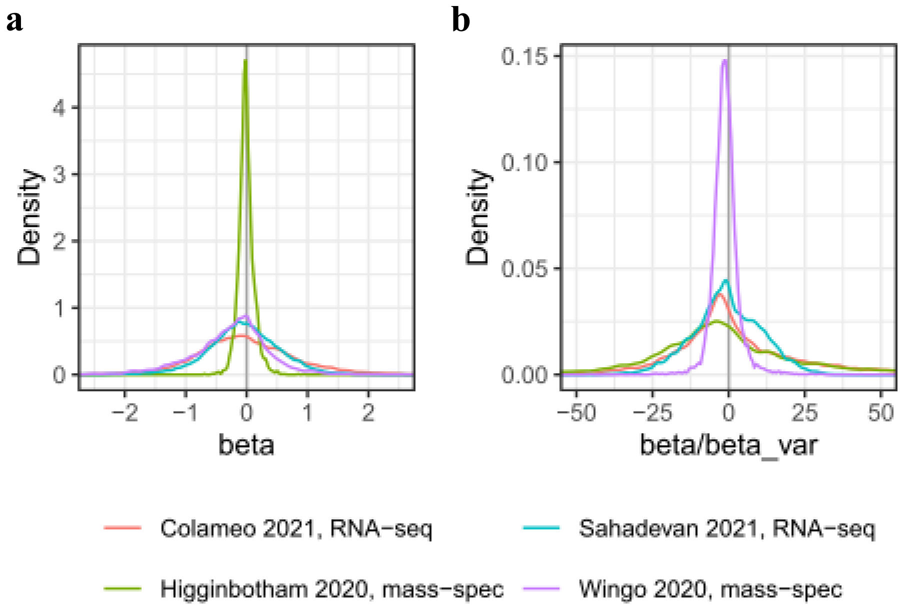

Supplementary Figure 5. Characterization of real-world datasets that were used for ROC analyses. Related to Figure 3.

Unlike GOAT and GSEA, iDEA does not apply rank transformation to input data and thus has to deal with a variety of input data distributions. **a**) log2 foldchange (beta) distributions from 4 real-world datasets. **b**) Following the iDEA user manual, the variation of each input gene's beta (beta_var) was computed from the input gene list log2 foldchange and p-values (i.e. beta and beta_var are both required for typical iDEA analyses). The distributions of beta/beta_var ratios illustrates strong differences between datasets.

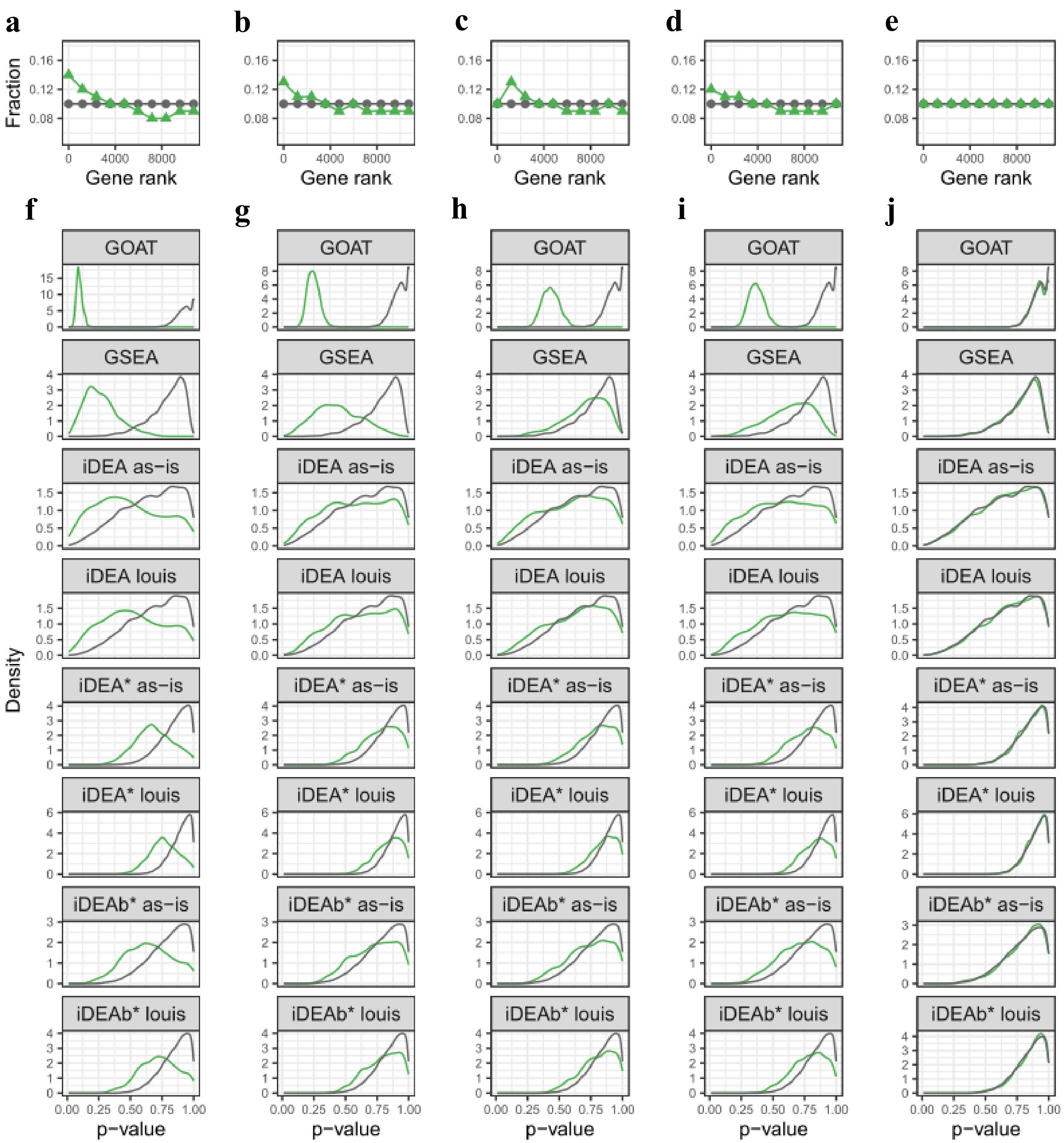

Supplementary Figure 6. Gene set p-value distributions from various simulated gene set collections, based on the Colameo RNA-sequencing dataset. Related to Figure 3.

Data shown here are from the same simulations as shown in Figure 3. **a-d**) Templates used to simulate 10 000 null gene sets (grey dots) and 1000 foreground gene sets (green triangles), each containing 100 genes. The x-axis represents gene ranks in the input gene list (Colameo et al. RNA-seq dataset). The y-axis shows the relative proportion of genes drawn from each of 10 bins. The null gene sets are always drawn from a uniform distribution whereas the foreground gene sets are drawn from various enrichment patterns (i.e. many~few top-ranked genes). **e**) As a control, draw foreground gene sets from a uniform distribution. **f-j**) For each gene set enrichment algorithm, show the respective gene set p-value distributions for both classes of gene sets (null gene sets in grey, foreground in green). Panels in each column match the simulated dataset designs from the panel in the top row (**a-e**). iDEA as-is: iDEA without louis p-value correction, iDEA*: iDEA with rescaled beta_var, iDEAb*: iDEA alternative model (testing beta only) with rescaled beta.

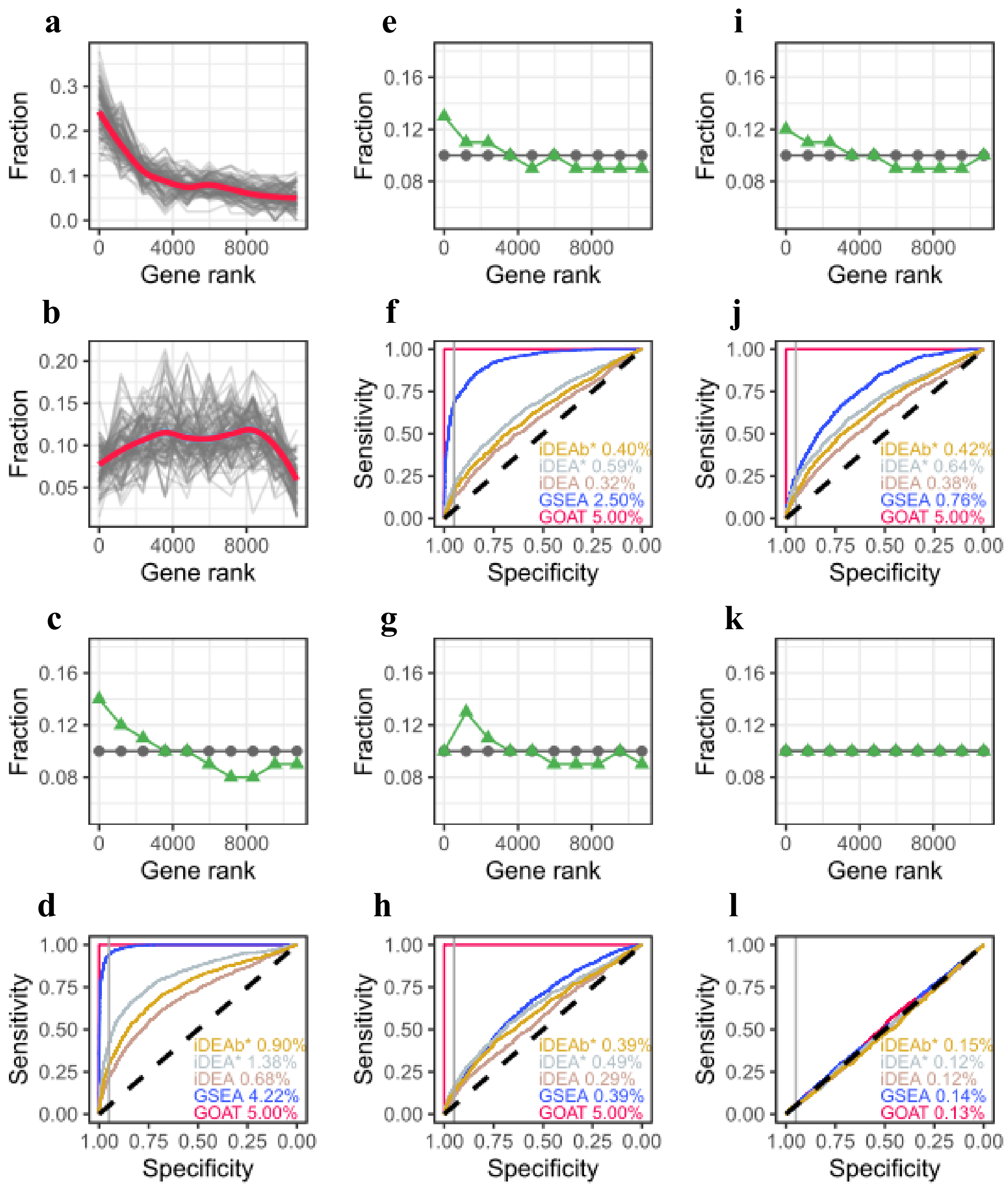

Supplementary Figure 7. All ROC simulations using the Colameo RNA-sequencing dataset as input gene list. Related to Figure 3.

Compared to Figure 3, we here included additional enrichment patterns that are shown in panels g,h and i,j. Panels from Figure 3 are here repeated for the convenience of easier comparison to repeated analyses with alternative input gene sets that are shown in Supplementary Figures 8-10. The gene set p-values that were computed by each method, which are the input to the ROC shown here, are shown in Supplementary Figure 6.

a,b) Gene rank distribution for constituents from the top (a) and bottom (b) 10% most significant downregulated GO terms (containing 50-500 genes) as detected by GSEA. **c,e,g,i,k**) Templates used to simulate 10000 null gene sets (grey dots) and 1000 foreground gene sets (green triangles). Values on the y-axis represent the proportion of random genes drawn from a respective bin in the input gene list (x-axis) to generate a gene set of 100 genes. **d,g,h,j,l**) Gene set enrichment analyses were applied to synthetic gene sets in the preceding figure panel. The ROC curves reflect the sensitivity at which respective methods assign a smaller p-value to foreground gene sets, enriched for top-ranked genes, as compared to null gene sets that were drawn from uniform distributions. **k,l**) As a control, draw foreground gene sets from a uniform distribution. Inset values for

each method represent the partial Area Under Curve (pAUC) at 95% specificity (vertical grey line). iDEA*: iDEA with rescaled beta_var, iDEAb*: iDEA alternative model (testing beta only) with rescaled beta.

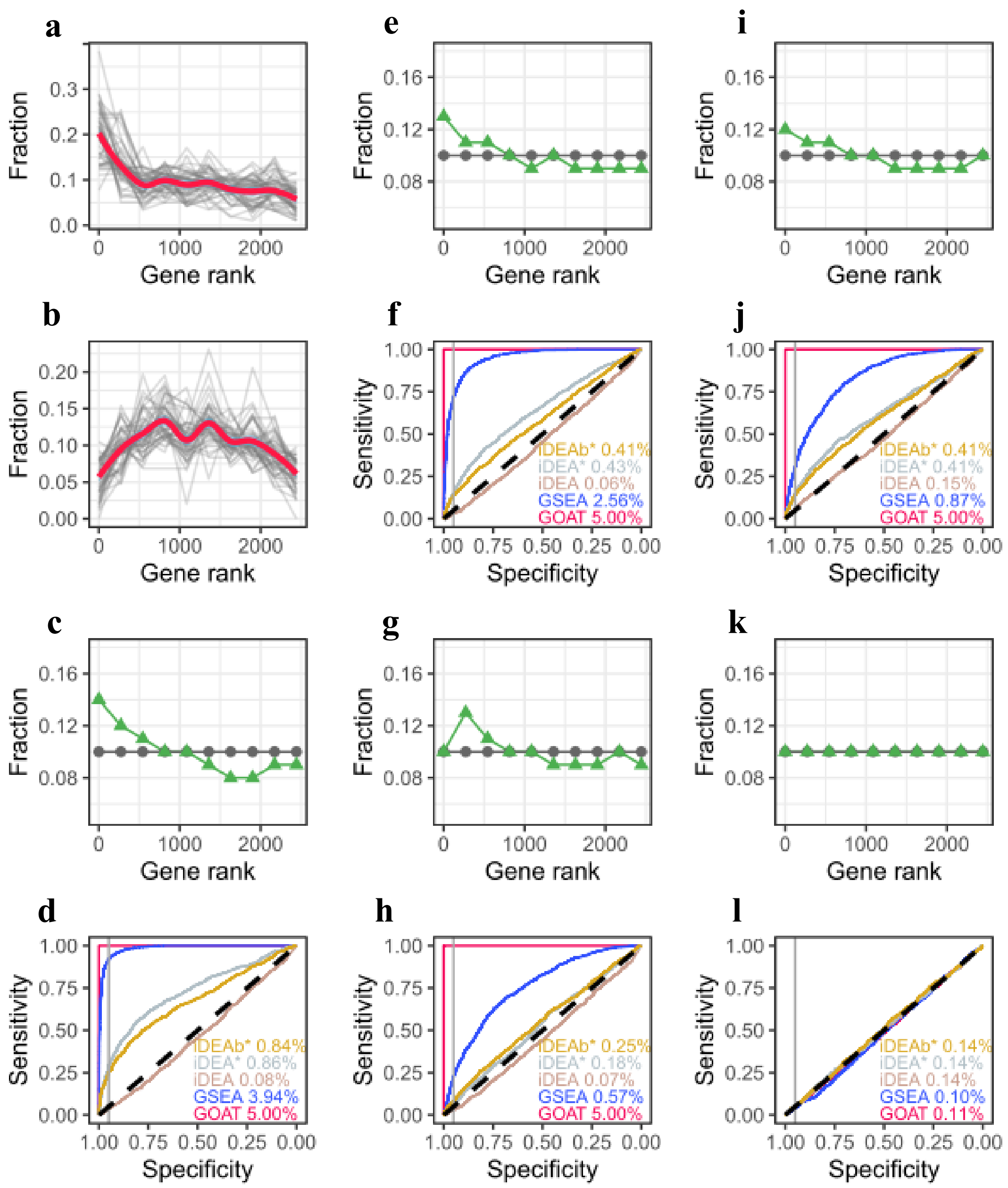

Supplementary Figure 8. Analogous to Supplementary Figure 7, but here using the Higginbotham mass spectrometry dataset as input. Related to Figure 3.

a,b) Gene rank distribution for constituents from the top (a) and bottom (b) 10% most significant downregulated GO terms (containing 50-500 genes) as detected by GSEA. **c,e,g,i,k**) Templates used to simulate 10000 null gene sets (grey dots) and 1000 foreground gene sets (green triangles). Values on the y-axis represent the proportion of random genes drawn from a respective bin in the input gene list (x-axis) to generate a gene set of 100 genes. **d,g,h,j,l**) Gene set enrichment analyses were applied to synthetic gene sets in the preceding figure panel. The ROC curves reflect the sensitivity at which respective methods assign a smaller p-value to foreground gene sets, enriched for top-ranked genes, as compared to null gene sets that were drawn from uniform distributions. **k,l**) As a control, draw foreground gene sets from a uniform distribution. Inset values for each method represent the partial Area Under Curve (pAUC) at 95% specificity (vertical grey line). iDEA*: iDEA with rescaled beta_var, iDEAb*: iDEA alternative model (testing beta only) with rescaled beta.

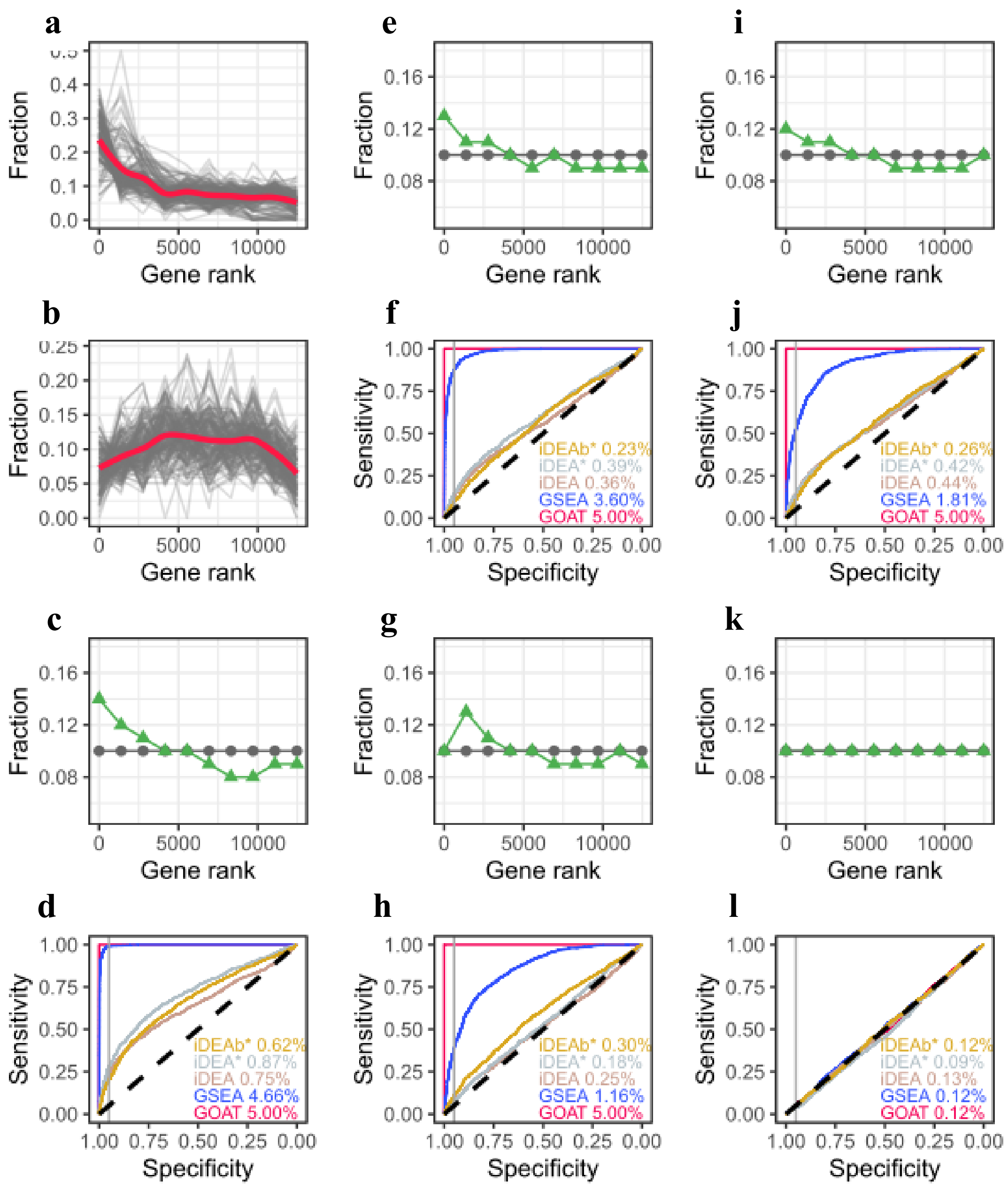

Supplementary Figure 9. Analogous to Supplementary Figure 7, but here using the Sahadevan RNA-sequencing dataset as input. Related to Figure 3.

a,b) Gene rank distribution for constituents from the top (a) and bottom (b) 10% most significant downregulated GO terms (containing 50-500 genes) as detected by GSEA. **c,e,g,i,k**) Templates used to simulate 10000 null gene sets (grey dots) and 1000 foreground gene sets (green triangles). Values on the y-axis represent the proportion of random genes drawn from a respective bin in the input gene list (x-axis) to generate a gene set of 100 genes. **d,g,h,j,l**) Gene set enrichment analyses were applied to synthetic gene sets in the preceding figure panel. The ROC curves reflect the sensitivity at which respective methods assign a smaller p-value to foreground gene sets, enriched for top-ranked genes, as compared to null gene sets that were drawn from uniform distributions. **k,l**) As a control, draw foreground gene sets from a uniform distribution. Inset values for each method represent the partial Area Under Curve (pAUC) at 95% specificity (vertical grey line). iDEA*: iDEA with rescaled beta_var, iDEAb*: iDEA alternative model (testing beta only) with rescaled beta.

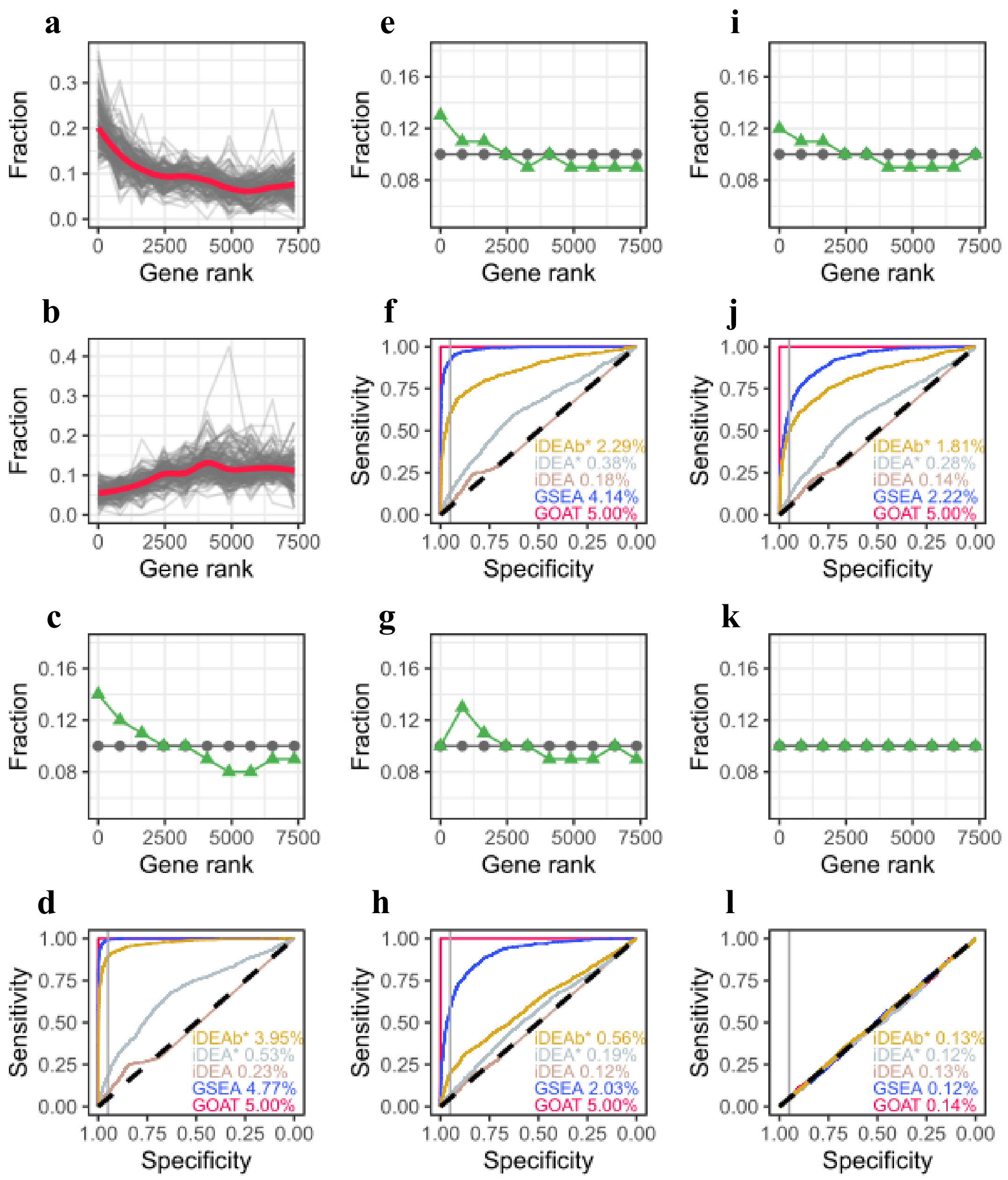

Supplementary Figure 10. Analogous to Supplementary Figure 7, but here using the Wingo mass spectrometry dataset as input. Related to Figure 3.

a,b) Gene rank distribution for constituents from the top (a) and bottom (b) 10% most significant downregulated GO terms (containing 50-500 genes) as detected by GSEA. **c,e,g,i,k**) Templates used to simulate 10000 null gene sets (grey dots) and 1000 foreground gene sets (green triangles). Values on the y-axis represent the proportion of random genes drawn from a respective bin in the input gene list (x-axis) to generate a gene set of 100 genes. **d,g,h,j,l**) Gene set enrichment analyses were applied to synthetic gene sets in the preceding figure panel. The ROC curves reflect the sensitivity at which respective methods assign a smaller p-value to foreground gene sets, enriched for top-ranked genes, as compared to null gene sets that were drawn from uniform distributions. **k,l**) As a control, draw foreground gene sets from a uniform distribution. Inset values for each method represent the partial Area Under Curve (pAUC) at 95% specificity (vertical grey line). iDEA*: iDEA with rescaled beta_var, iDEAb*: iDEA alternative model (testing beta only) with rescaled beta.

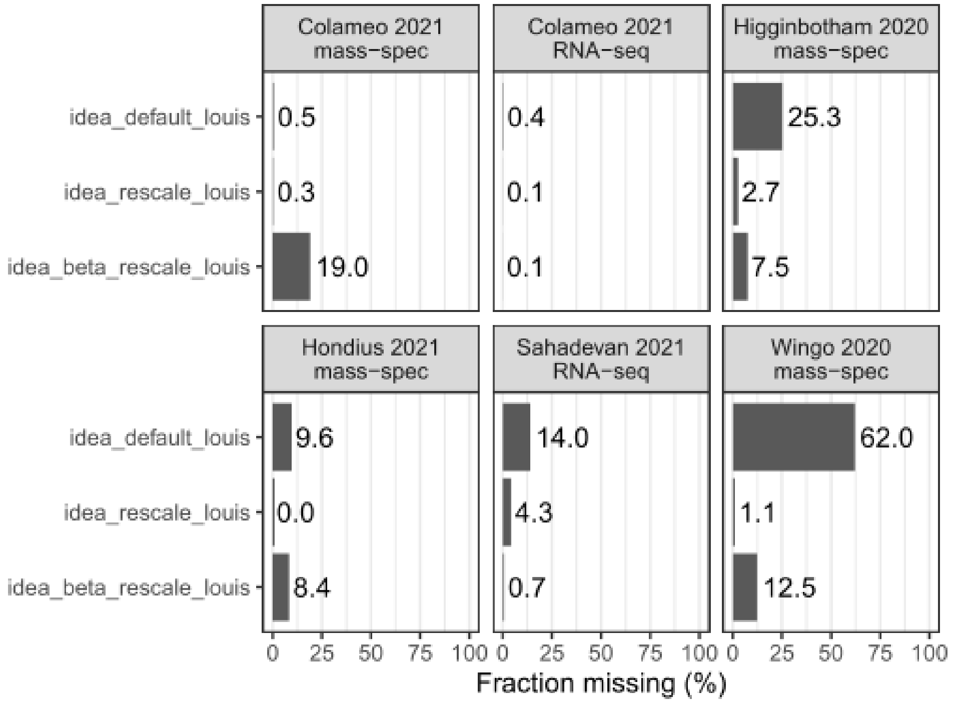

Supplementary Figure 11. Proportion of gene sets where iDEA did not return a valid p-value. Related to Figure 4.

Three variants of iDEA (y-axis) were used to perform GO analysis of 6 real-world datasets (panels) in order to quantify how many gene sets resulted in errors (x-axis).
"idea_default_louis" represents the default workflow for iDEA (per instructions in the iDEA manual); estimate variation of gene log2fc values from input gene list p-values, apply iDEA with default parameters, finally correct gene set p-values with the louis method.
"idea_rescale_louis" represents an alternative workflow that we implemented, which rescales the estimated gene log2fc variations such that their distribution approximates a gamma distributions with shape=2 and scale=0.5. This greatly reduced the number of errors returned by iDEA.
"idea_rescale_louis" represents another alternative workflow, where we rescale the log2fc values from the input gene list by their median absolute deviation and then apply iDEA with "modelVariant" set to TRUE such that iDEA directly models the (now standardized) log2fc values.

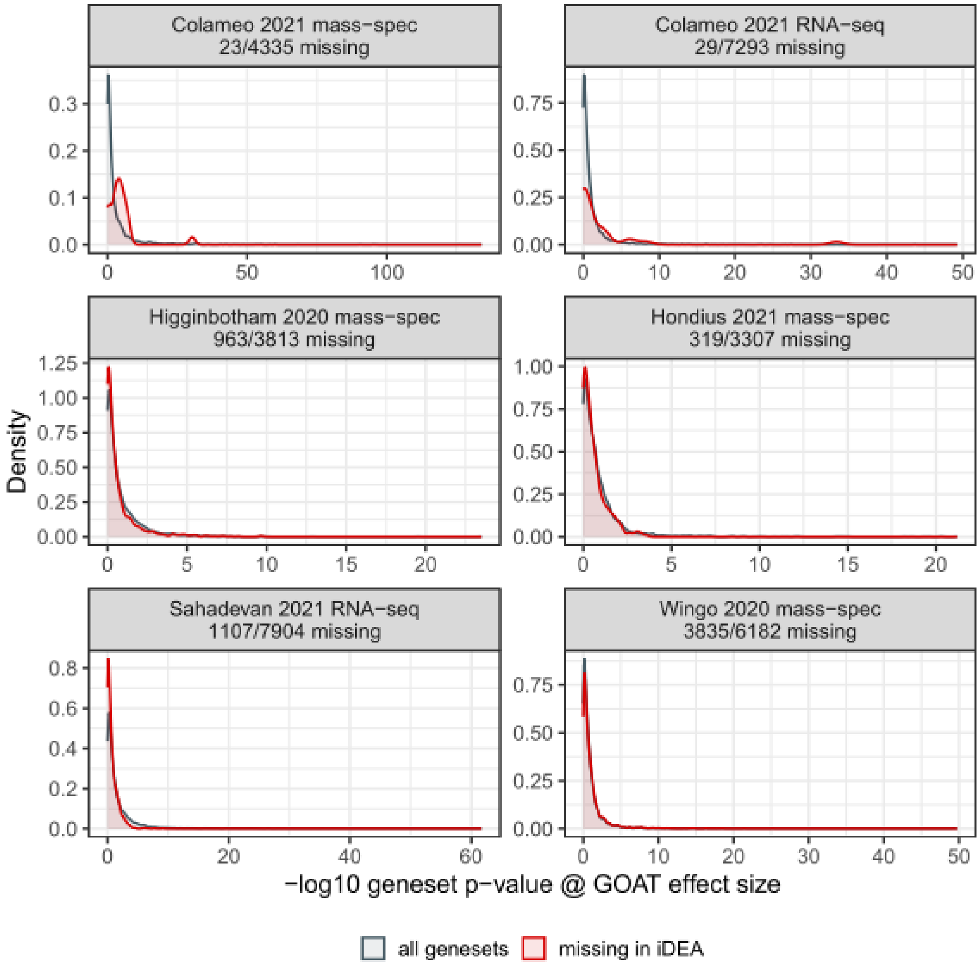

Supplementary Figure 12. Characterization of missing data in iDEA. Related to Figure 4.

The iDEA algorithm produced gene set p-values for only a subset of gene sets in most studies we benchmarked. The title of each panel reflects the respective dataset and the number of gene sets where an iDEA p-value was missing. Note that for some datasets, the rate of missingness is very high. The plots describe the -log10 gene set p-value distributions obtained with GOAT (grey line), with the (distribution of the) subset of gene sets that are missing in iDEA shown in red. These distributions were generally similar, i.e. missingness in iDEA did not bias for or against gene sets that are highly enriched according to GOAT.

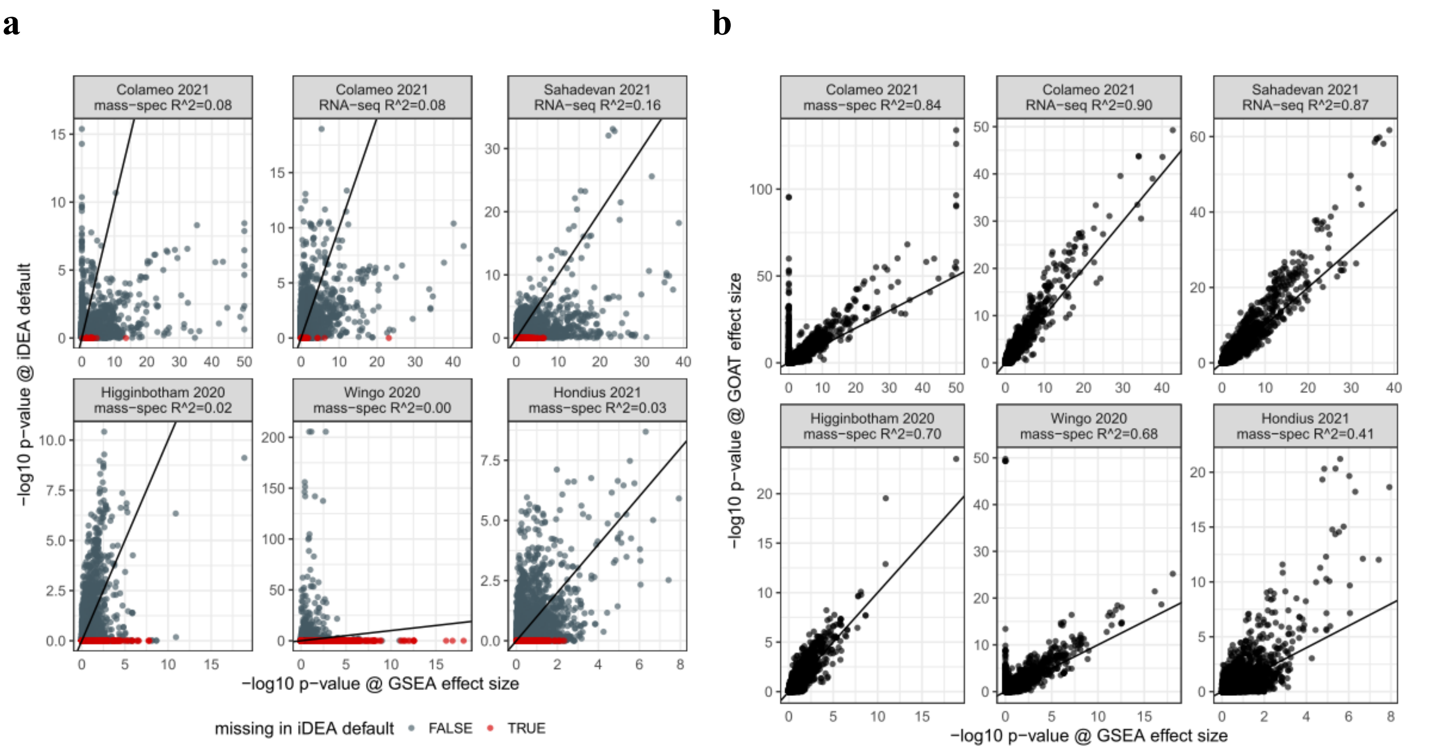

Supplementary Figure 13. Comparison of GSEA gene set p-values to iDEA and GOAT. Related to Figure 4.

a) Gene set -log10 p-values (without multiple testing adjustment) for GSEA and iDEA ("louis" p-values) showed a lack of agreement between both methods. **b**) Comparing GSEA and GOAT, gene sets with a strong p-value in GSEA showed a similar trend in GOAT. The similarity between gene set enrichment methods was quantified by computing an R^2 from the subset of gene sets that is among the top 25% results in either method (and has a p-value in both methods). The black line represents the diagonal.

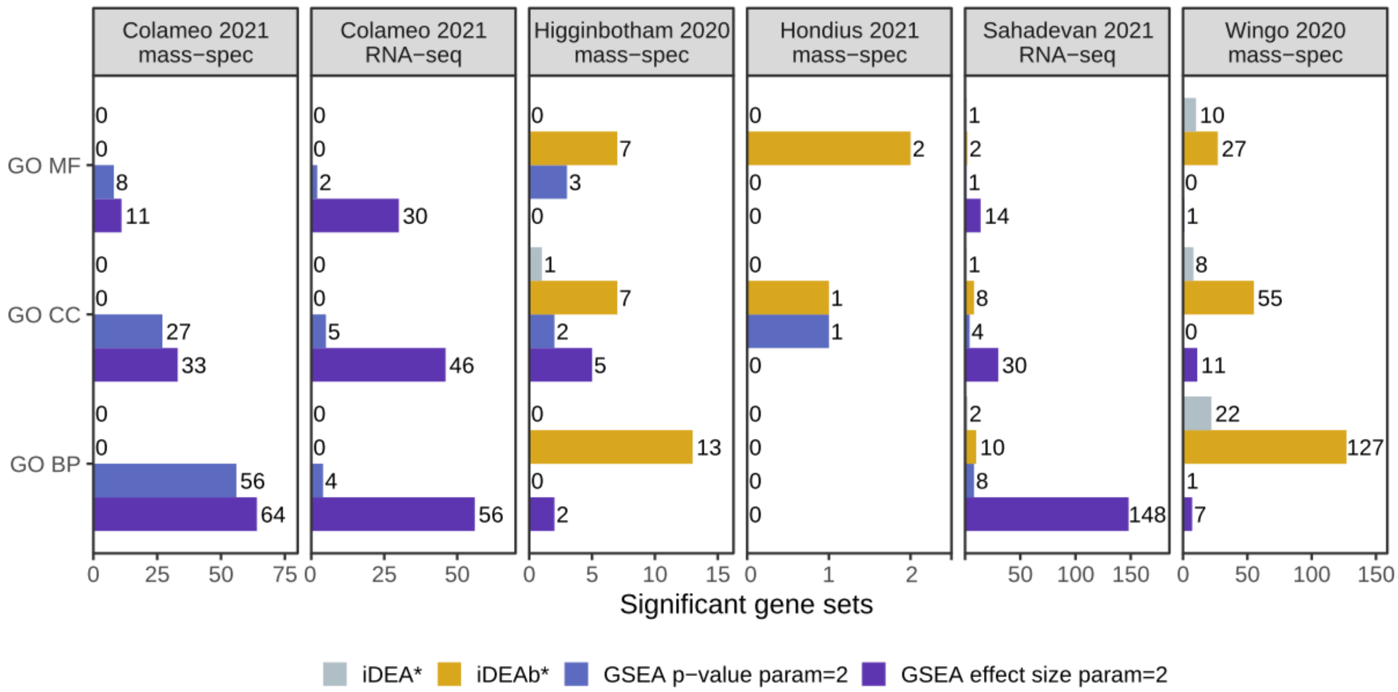

Supplementary Figure 14. Application of alternative GSEA and iDEA configurations to real-world data. Related to Figure 4.

Application of alternative gene set enrichment methods to various real-world datasets; an extension of Figure 4. iDEA*: iDEA with rescaled beta_var, iDEAb*: iDEA alternative model (testing beta only) with rescaled beta. GSEA (fGSEA implementation) was applies with either a gene list of p-values or a gene list with effect sizes as input, here specifically using the alternative setting “gseaParam = 2” which subsequently causes fGSEA to transforms the gene scores/weights to score^2 prior to gene set enrichment calculation.

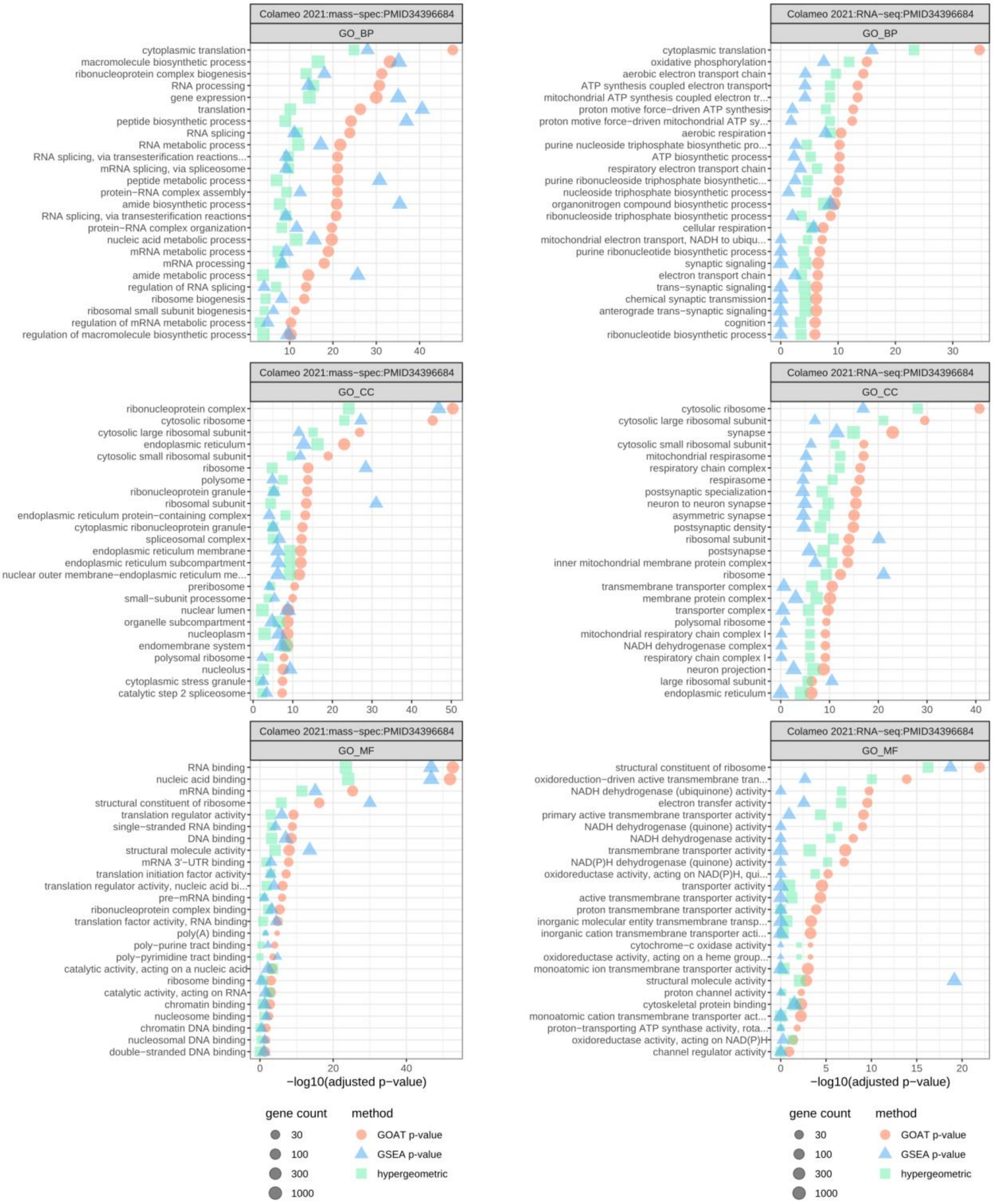

Supplementary Figure 15. Comparison of GOAT top-hits with GSEA and ORA in application to real-world data. Related to Figure 4.

GOAT (orange circle), GSEA (blue triangle) and ORA (green square) were applied to gene lists with p-values from the Colameo et al. mass spectrometry and RNA-sequencing studies. The 25 gene sets with the strongest p-value obtained from GOAT are shown for each GO domain. The respective GSEA and ORA results generally show the same trend of enrichment albeit with less significance. GO_MF, GO_CC and GO_BP represent the respective Gene Ontology domains Molecular Functions, Cellular Components and Biological Processes. The x-axis shows gene set p-values after Bonferroni adjustment on -log10 scale.
